# Bis(8-hy­droxy­quinolinium) naphthalene-1,5-di­sulfonate tetra­hydrate

**DOI:** 10.1107/S2414314624005704

**Published:** 2024-06-18

**Authors:** Yusufjon Eshkobilovich Nazarov, Khayit Khudaynazarovich Turaev, Bekmurod Khurramovich Alimnazarov, Jabbor Ruziboevich Suyunov, Gulnora Abdurakhmonovna Umirova, Bakhtiyar Tulyaganovich Ibragimov, Jamshid Mengnorovich Ashurov

**Affiliations:** a Termez State University, Barkamol Avlod Street 43, Termez City, Uzbekistan; b Institute of Bioorganic Chemistry, Academy of Sciences of Uzbekistan, 100125, M. Ulugbek Str 83, Tashkent, Uzbekistan; Vienna University of Technology, Austria

**Keywords:** naphthalene-1,5-di­sulfonate, 8-hy­droxy­quinoline, crystal structure, hydrogen bonds, organic salt

## Abstract

The inter­action between 8-hy­droxy­quinoline (8HQ, C_9_H_7_NO) and naphthalene-1,5-di­sulfonic acid (H_2_NDS, C_10_H_8_O_6_S_2_) in aqueous media results in the formation of the salt hydrate bis­(8-hy­droxy­quinolinium) naphthalene-1,5-di­sulfonate tetra­hydrate, 2C_9_H_8_NO^+^·C_10_H_6_O_6_S_2_^2−^·4H_2_O. The asymmetric unit comprises one protonated 8HQ^+^ cation, half of an NDS^2–^ dianion symmetrically disposed around a center of inversion, and two water mol­ecules. Within the crystal structure, these components are organized into chains along the [010] and [10] directions through O—H⋯O and N—H⋯O hydrogen-bonding inter­actions, forming a di-periodic network parallel to (101). Additional stabilizing inter­actions such as C—H⋯O, C—H⋯π, and π–π inter­actions extend this arrangement into a tri-periodic network structure

## Abstract 2

The crystal structure of the organic salt features classical hydrogen-bonding inter­actions involving the 8-hy­droxy­quinolinium cation, the naphthalene-1,5-di­sulfonate anion and the two water mol­ecules of crystallization.

## Graphical abstract

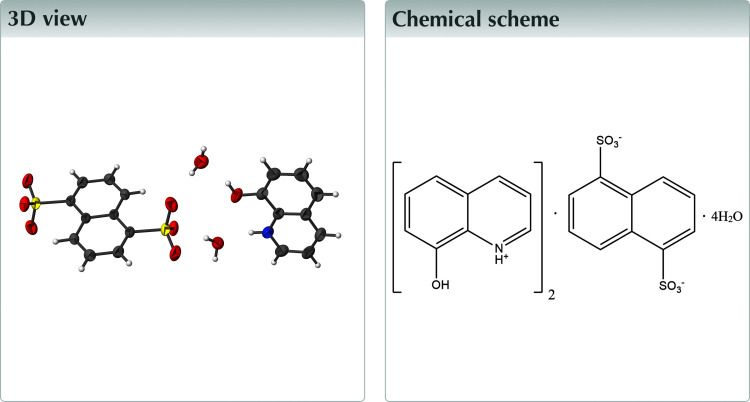

## Structure description

8-Hy­droxy­quinoline (8HQ, C_9_H_7_NO, H*L*), known also as oxine, is a bidentate chelating agent. It forms three species: H_2_*L*^+^, H*L* and *L*^−^. 8HQ bearing a hetero-nitro­gen atom (p*K*a = 10.8) and the 8-substituted phenol group (p*K*a = 4.9) is a good organic acid–base adduct conformer and has been reported to form supra­molecularly organized compounds with acidic counter parts under formation of multiple hydrogen bonds (Smith *et al.*, 2003 ▸). 8HQ is used in analytical chemistry for the qu­anti­tative determination of metal ions because the resulting complexes are insoluble in water. The aluminium complex (Cölle *et al.*, 2002 ▸; Katakura & Koide, 2006 ▸) is a common component of organic light-emitting diodes (OLEDs). Substituents on the quinoline ring result in compounds with luminescence properties (Montes *et al.*, 2006 ▸). In its photo-induced excited state, 8HQ converts to zwitterionic isomers, in which the hydrogen atom is transferred from oxygen to nitro­gen (Bardez *et al.*, 1997 ▸). The complexes as well as the heterocycle itself exhibit anti­septic, disinfectant, and pesticidal properties (Phillips *et al.*, 1956 ▸) and functions as a transcription inhibitor (Wen *et al.*, 2023 ▸). Its solution in alcohol is used in liquid bandages. It once was of inter­est as an anti-cancer drug (Zhu *et al.*, 2017 ▸; Fouda 2017 ▸). The roots of the invasive plant *Centaurea diffusa* release 8HQ, which has a negative effect on plants that have not co-evolved with it (Vivanco *et al.*, 2004 ▸).

1,5-Naphthalene­disulfonic acid (H_2_NDS, Armstrong acid, C_10_H_8_O_6_S_2_) is a white-to-yellowish solid that is soluble in water (1030 g l^−1^). It is used in the production of dyes, pigments, and other industrial chemicals. It also functions as a chelating and complexing agent, which is used in various applications such as water treatment, analytical chemistry, and mineral processing (Arslan-Alaton *et al.*, 2008 ▸). H_2_NDS does not demonstrate a definite biological activity. Complexes derived from H_2_NDS are of inter­est in supra­molecular chemistry due to their ability to form complex hydrogen-bonded systems because the sulfonate group can accept up to six hydrogen bonds.

Preparation and structural characterization of organic salts on basis of these two simple compounds is of inter­est for supra­molecular and analytical chemistry (Oh *et al.*, 2020 ▸; Chen *et al.*, 2022 ▸). In our previous works (Suyunov *et al.*, 2023*a* ▸,*b* ▸,*c* ▸), we reported on H_2_NDS and its salts involving nickel(II) and cadmium(II). In the current work, we report on preparation and mol­ecular and crystal structures of a proton-transfer salt, 2(8HQ)^+^·NDS^2–^·4H_2_O.

**Figure 1:**
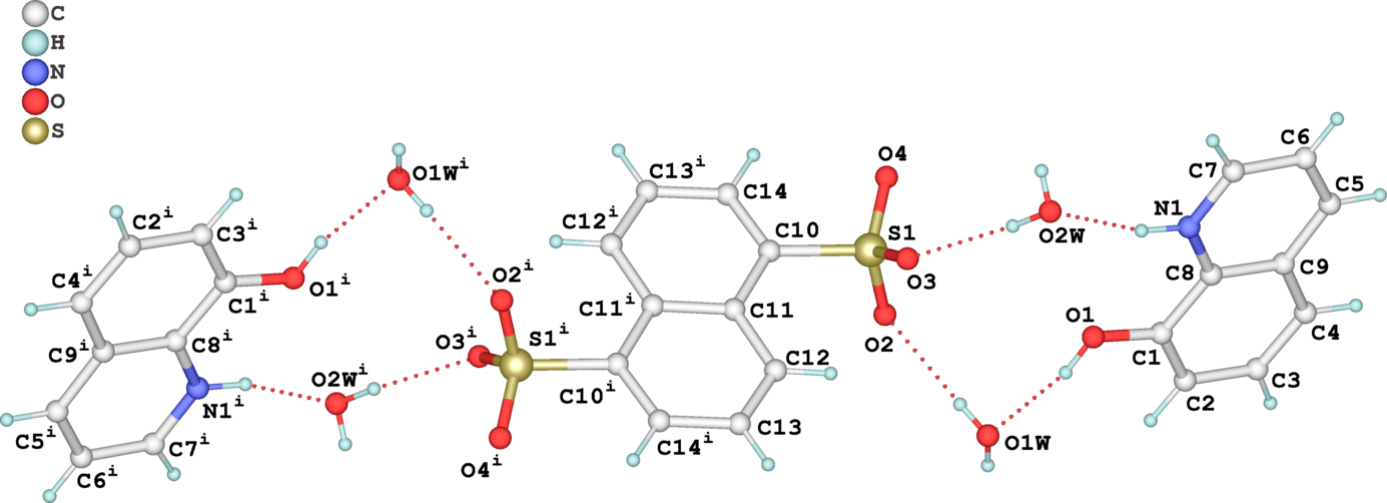
The structures of the mol­ecular entities in the title salt, showing the atom-labelling scheme and displacement ellipsoids drawn at the 50% probability level. H atoms are shown as spheres of arbitrary radius and hydrogen bonds are shown as dashed lines. [Symmetry code: (i) −*x*, 1 − *y*, 1 - *z.*]

The asymmetric unit of the title compound consists of one 8HQ^+^ cation, half of an NDS^2–^ anion, and two water mol­ecules of crystallization, resulting in a supra­molecular associate with a 2:1:4 cation-anion-water composition. The sulfonic acid (SO_3_H) groups of H_2_NDS are deprotonated, with the hydrogen atoms transferred to the nitro­gen atom of an 8HQ^+^ cation, and the NDS^2−^ dianion exhibits inversion symmetry, with the inversion center located at the midpoint of the C11—C11^i^ [symmetry code: (i) −*x*, 1 − *y*, 1 − *z*] bond in the naphthalene ring system (Fig. 1 ▸). A similar salt with composition 2C_9_H_8_NO^+^·C_10_H_6_O_6_S_2_^2–^·2H_2_O was previously reported (Jin *et al.*, 2014 ▸), the main difference being the presence of only two water mol­ecules and ortho­rhom­bic symmetry (space group *Pbca*) compared to four water mol­ecules and monoclinic symmetry (space group *P*2_1_/*n*) for the title salt. In the cation of the title salt, the angle around the protonated N atom [C7—N1—C8 = 122.67 (13)°] is approximately 1° less than the corresponding angle in the study of the dihydrate [123.5 (3)°]. In the title salt, the anions exhibit two distinct orientations, with the angle between their planes being 33.37 (7)°. The cations are oriented in a single direction, forming angles of 71.66 (8) and 75.80 (9)° with the planes of the anions. The naphthalene ring system exhibits typical bond lengths and angles, with C—C bond lengths ranging from 1.362 (2) to 1.431 (2) Å, and C—C—C angles in the range 117.91 (14) to 123.05 (12)°. The hy­droxy­quinoline and naphthalene fragments are coplanar with r.m.s deviations of 0.0162 (14) Å and 0.0112 (13) Å.

**Figure 2:**
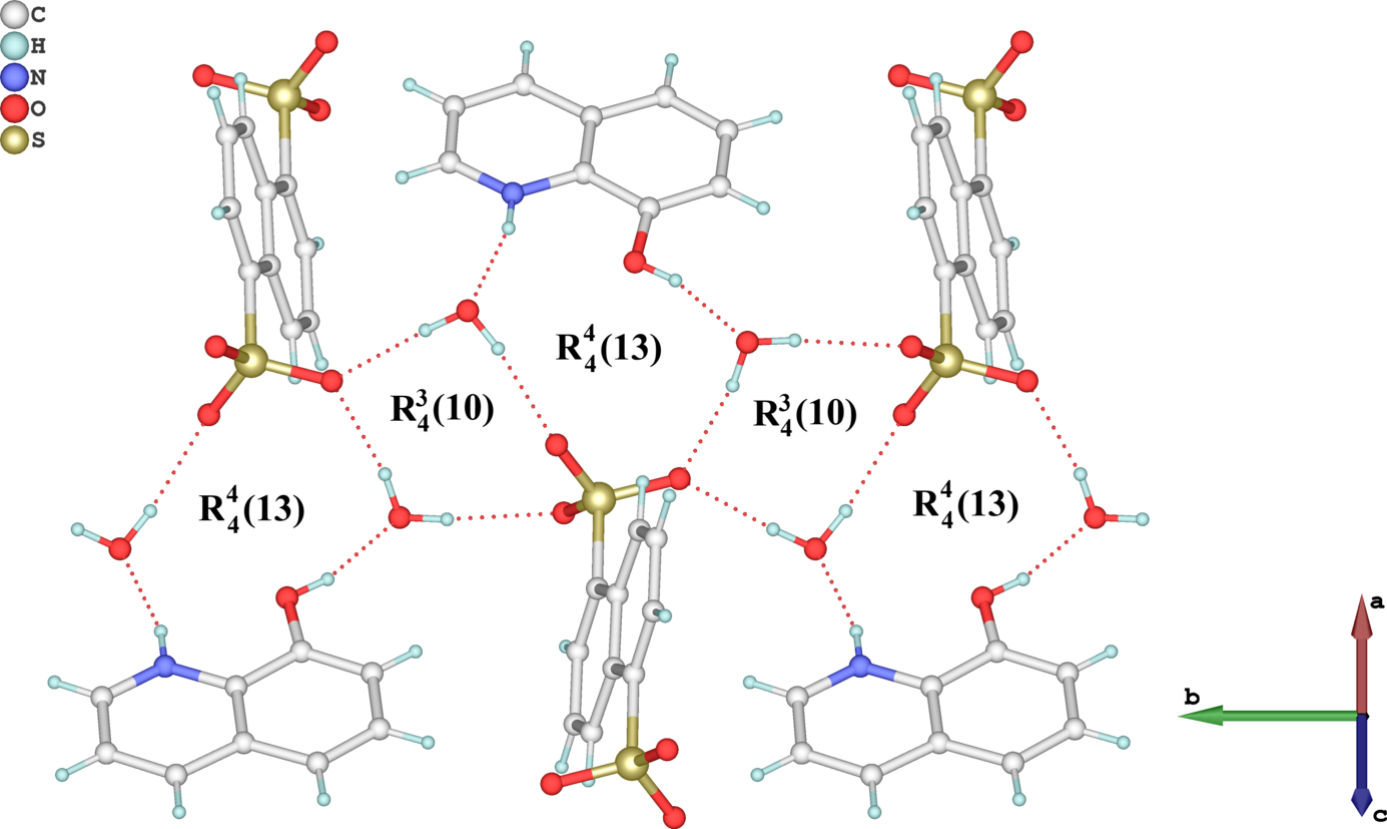
The formation of O—H⋯O and N—H⋯O hydrogen bonds (dashed red lines) in the crystal structure, leading to 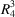
(10) and 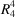
(13) graph-set motifs.

**Figure 3:**
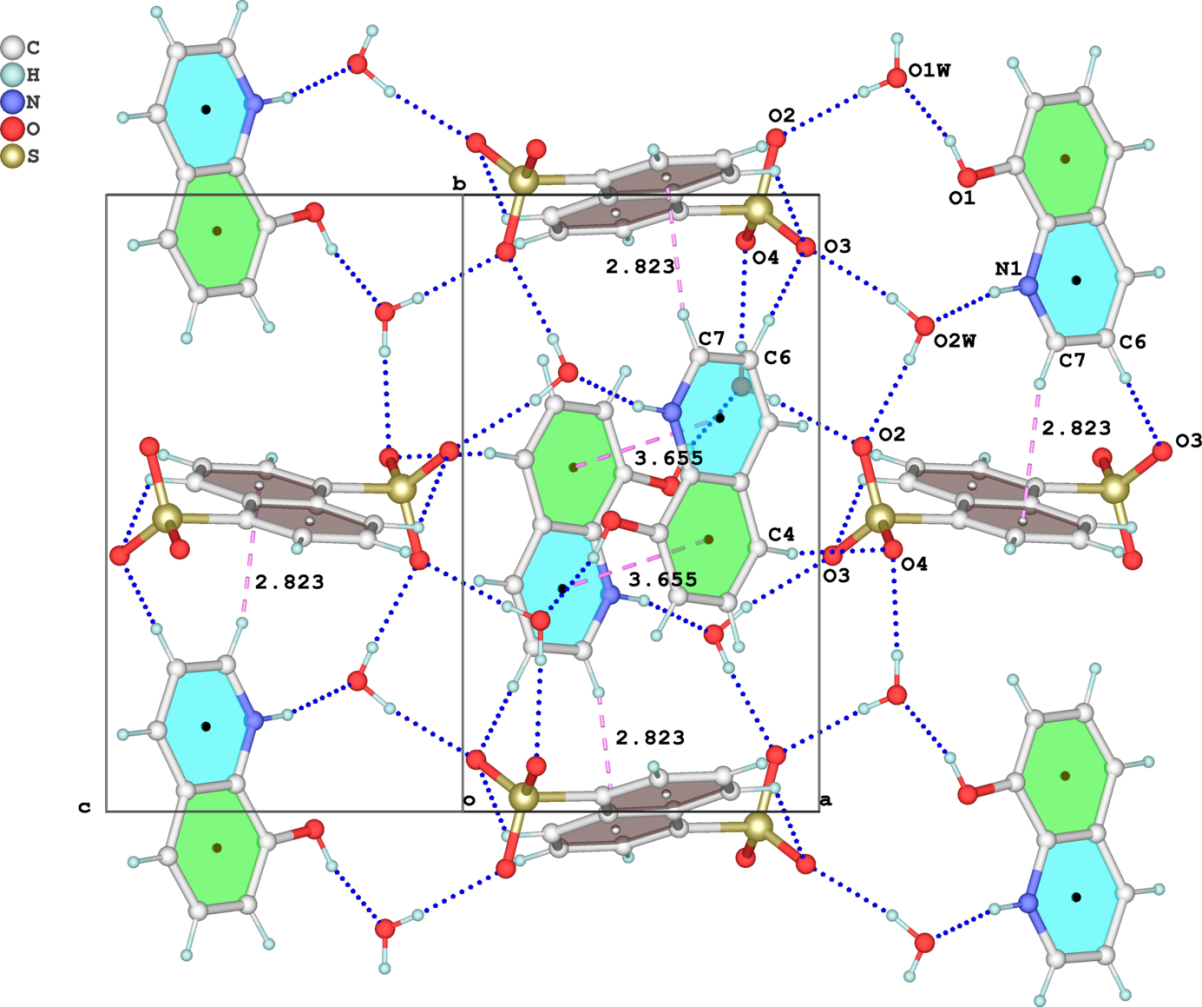
The crystal packing of the title salt in a view along [101]. O—H⋯O, N—H⋯O, and C—H⋯O hydrogen bonds are shown as dashed blue lines, and C—H⋯π and π–π inter­actions as dashed pink lines.

In the crystal, the 8HQ^+^ cation, the NDS^2–^ anion, and the water mol­ecules are connected *via* classical O—H⋯O and N—H⋯O hydrogen bonds (Table 1 ▸) with graph-set motifs of 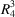
(10) and 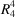
(13), which link the components into chains extending parallel to [010], as illustrated in Fig. 2 ▸. The SO_3_^−^ group on one side of the anion participates in the formation of these chains. The symmetry-related second SO_3_^−^ group also participates in hydrogen bonding under the formation of a second infinite chain parallel to [10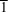] connecting with the previous chains *via* C—H⋯π inter­actions (where *Cg* are the centroids of the naphthalene rings, Table 1 ▸) and C(π)⋯C,N(π) weak inter­molecular contacts [*Cg*⋯*Cg* distance = 3.6547 (9) Å, slippage 1.248 Å], forming sheets parallel to (101) (Fig. 3 ▸). These sheets are linked through additional weak C—H⋯O inter­actions into a tri-periodic network structure. Due to steric hindrance of the sulfonate groups, the nearest centroid separation between naphthalene rings is 5.264 (3) Å, suggesting no π–π stacking between these moieties.

A search of the Cambridge Structural Database (CSD, version 5.45, updated November 2023; Groom *et al.*, 2016 ▸) revealed that the crystal structure of 8HQ alone has been determined eleven times, while thirteen reports are related to mol­ecular complexes, and 71 crystals are organic salts where the nitro­gen atom of 8HQ is protonated. In the case of 1,5-NDSA, 225 crystals are organic salts of 1,5-NDSA in the dianionic form, One compound (FIVFOI01; Du *et al.*, 2019 ▸) is a complex with 1,5-NDSA in the monoanionic form, and four crystals are mol­ecular complexes (SAHRIG, Singh *et al.*, 2021 ▸; SATBEX, Liu *et al.*, 2017 ▸; VEGHUN, Cunha *et al.*, 2017 ▸; WEZGAN, Xu *et al.*, 2023 ▸) with neutral sulfo-acid mol­ecules.

## Synthesis and crystallization

The title compound was obtained by the addition of 1,5-naphthalene­disulfonate acid (0.288 g, 1 mmol) to a solution of 8-hy­droxy­quinoline (0.176 g, 2 mmol) in water, in the stoichiometric ratio 1:2. Good-quality single crystals were obtained by slow evaporation after four days (yield: 60%).

## Refinement

Crystal data, data collection and structure refinement details are summarized in Table 2 ▸.

## Supplementary Material

Crystal structure: contains datablock(s) I. DOI: 10.1107/S2414314624005704/wm4216sup1.cif

Structure factors: contains datablock(s) I. DOI: 10.1107/S2414314624005704/wm4216Isup2.hkl

Supporting information file. DOI: 10.1107/S2414314624005704/wm4216Isup3.cml

CCDC reference: 2256736

Additional supporting information:  crystallographic information; 3D view; checkCIF report

## Figures and Tables

**Table 1:** Hydrogen-bond geometry (Å, °) *Cg*1 and *Cg*2 are the centroids of the C10/ C11/C11′–C13′/C14 and C11–C13/C14′/C10′/C11′ rings, respectively, where primed atoms are related by the symmetry operation −*x*, 1 − *y*, 1 − *z*.

*D*—H⋯*A*	*D*—H	H⋯*A*	*D*⋯*A*	*D*—H⋯*A*
O2*W*—H2*WA*⋯O2^i^	0.85	1.98	2.8239 (16)	176
O2*W*—H2*WB*⋯O3	0.85	2.02	2.8610 (15)	168
O1*W*—H1*WA*⋯O2	0.85	1.98	2.8150 (17)	169
O1*W*—H1*WB*⋯O4^ii^	0.85	2.05	2.8806 (18)	166
O1—H1⋯O1*W*	0.82 (1)	1.84 (1)	2.6390 (16)	165 (2)
N1—H1*A*⋯O2*W*	0.87 (1)	1.89 (1)	2.7347 (18)	164 (2)
C6—H6⋯O3^iii^	0.93	2.46	3.3153 (19)	154
C4—H4⋯O4^iv^	0.93	2.45	3.352 (2)	165
C14—H14⋯O1^v^	0.93	2.57	3.3173 (18)	137
C12—H12⋯O3	0.93	2.46	3.0439 (17)	121
C7—H7⋯*Cg*1^i^	0.93	2.82	3.6125 (17)	144
C7—H7⋯*Cg*2^iii^	0.93	2.82	3.6125 (17)	144

Symmetry codes: (i) 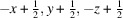; (ii) 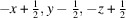; (iii) 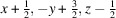; (iv) 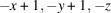; (v) 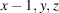.

**Table 2:** Experimental details

Crystal data	
Chemical formula	2C_9_H_8_NO^+^·C_10_H_6_O_6_S_2_^2−^·4H_2_O
*M*_r_	650.66
Crystal system, space group	Monoclinic, *P*2_1_/*n*
Temperature (K)	290
*a*, *b*, *c* (Å)	7.55855 (8), 12.16674 (13), 16.00467 (17)
β (°)	94.7152 (10)
*V* (Å^3^)	1466.86 (3)
*Z*	2
Radiation type	Cu *K*α
μ (mm^−1^)	2.25
Crystal size (mm)	0.32 × 0.3 × 0.28

Data collection	
Diffractometer	XtaLAB Synergy, Single source at home/near, HyPix3000
Absorption correction	Multi-scan (*CrysAlis PRO*; Rigaku OD, 2022 ▸)
*T*_min_, *T*_max_	0.820, 1.000
No. of measured, independent and observed [*I* > 2σ(*I*)] reflections	14094, 2841, 2621
*R*_int_	0.026
(sin θ/λ)_max_ (Å^−1^)	0.615

Refinement	
*R*[*F*^2^ > 2σ(*F*^2^)], *wR*(*F*^2^), *S*	0.031, 0.089, 1.05
No. of reflections	2841
No. of parameters	214
No. of restraints	2
H-atom treatment	H atoms treated by a mixture of independent and constrained refinement
Δρ_max_, Δρ_min_ (e Å^−3^)	0.20, −0.30

Computer programs: *CrysAlis PRO* (Rigaku OD, 2022 ▸), *SHELXT* (Sheldrick, 2015*a* ▸), *SHELXL* (Sheldrick, 2015*b* ▸), *OLEX2* (Dolomanov *et al.*, 2009 ▸) and *publCIF* (Westrip, 2010 ▸).

## full crystallographic data

### Bis(8-hydroxyquinolinium) naphthalene-1,5-disulfonate tetrahydrate Crystal data

**Table:**

2C_9_H_8_NO^+^·C_10_H_6_O_6_S_2_^2^^−^·4H_2_O	*F*(000) = 680
*M**_r_* = 650.66	*D*_x_ = 1.473 Mg m^−^^3^
Monoclinic, *P*2_1_/*n*	Cu *K*α radiation, λ = 1.54184 Å
*a* = 7.55855 (8) Å	Cell parameters from 9285 reflections
*b* = 12.16674 (13) Å	θ = 2.8–71.2°
*c* = 16.00467 (17) Å	µ = 2.25 mm^−^^1^
β = 94.7152 (10)°	*T* = 290 K
*V* = 1466.86 (3) Å^3^	Block, light yellow
*Z* = 2	0.32 × 0.3 × 0.28 mm

### Bis(8-hydroxyquinolinium) naphthalene-1,5-disulfonate tetrahydrate Data collection

**Table:**

XtaLAB Synergy, Single source at home/near, HyPix3000 diffractometer	2841 independent reflections
Radiation source: micro-focus sealed X-ray tube, PhotonJet (Cu) X-ray Source	2621 reflections with *I* > 2σ(*I*)
Mirror monochromator	*R*_int_ = 0.026
Detector resolution: 10.0000 pixels mm^-1^	θ_max_ = 71.4°, θ_min_ = 4.6°
ω scans	*h* = −9→9
Absorption correction: multi-scan (CrysAlisPro; Rigaku OD, 2022)	*k* = −14→14
*T*_min_ = 0.820, *T*_max_ = 1.000	*l* = −19→19
14094 measured reflections	

### Bis(8-hydroxyquinolinium) naphthalene-1,5-disulfonate tetrahydrate Refinement

**Table:**

Refinement on *F*^2^	Secondary atom site location: difference Fourier map
Least-squares matrix: full	Hydrogen site location: mixed
*R*[*F*^2^ > 2σ(*F*^2^)] = 0.031	H atoms treated by a mixture of independent and constrained refinement
*wR*(*F*^2^) = 0.089	*w* = 1/[σ^2^(*F*_o_^2^) + (0.0501*P*)^2^ + 0.303*P*] where *P* = (*F*_o_^2^ + 2*F*_c_^2^)/3
*S* = 1.05	(Δ/σ)_max_ < 0.001
2841 reflections	Δρ_max_ = 0.20 e Å^−^^3^
214 parameters	Δρ_min_ = −0.29 e Å^−^^3^
2 restraints	Extinction correction: SHELXL-2019/2 (Sheldrick 2015b), Fc^*^=kFc[1+0.001xFc^2^λ^3^/sin(2θ)]^-1/4^
Primary atom site location: intrinsic phasing	Extinction coefficient: 0.0023 (3)

### Bis(8-hydroxyquinolinium) naphthalene-1,5-disulfonate tetrahydrate Special details

**Table:**

Geometry. All esds (except the esd in the dihedral angle between two l.s. planes) are estimated using the full covariance matrix. The cell esds are taken into account individually in the estimation of esds in distances, angles and torsion angles; correlations between esds in cell parameters are only used when they are defined by crystal symmetry. An approximate (isotropic) treatment of cell esds is used for estimating esds involving l.s. planes.
Refinement. Hydrogen atoms attached to the N and O atoms were located from a difference-Fourier map and refined with bond-length restraints of 0.86 (1) Å and 0.82 (1) Å, respectively.

### Bis(8-hydroxyquinolinium) naphthalene-1,5-disulfonate tetrahydrate Fractional atomic coordinates and isotropic or equivalent isotropic displacement parameters (Å^2^)

**Table:**

	*x*	*y*	*z*	*U*_iso_*/*U*_eq_	
S1	0.12659 (4)	0.52366 (3)	0.29817 (2)	0.03571 (13)	
O2	0.16257 (16)	0.40840 (9)	0.28388 (7)	0.0539 (3)	
O3	0.28637 (14)	0.58461 (9)	0.32385 (6)	0.0451 (3)	
O4	0.02239 (15)	0.57423 (10)	0.22875 (6)	0.0525 (3)	
C10	−0.00953 (17)	0.52607 (10)	0.38419 (8)	0.0315 (3)	
C11	0.05915 (16)	0.49755 (10)	0.46738 (8)	0.0297 (3)	
C12	0.23846 (17)	0.46556 (11)	0.48773 (9)	0.0351 (3)	
H12	0.315658	0.460895	0.445520	0.042*	
C13	0.29860 (18)	0.44165 (13)	0.56821 (9)	0.0407 (3)	
H13	0.417007	0.422577	0.580589	0.049*	
C14	−0.18313 (18)	0.55446 (12)	0.36709 (9)	0.0379 (3)	
H14	−0.225612	0.571361	0.312413	0.045*	
O1	0.60496 (18)	0.47210 (9)	0.18830 (7)	0.0538 (3)	
N1	0.68475 (17)	0.64774 (10)	0.09816 (8)	0.0415 (3)	
C1	0.6624 (2)	0.45179 (12)	0.11258 (9)	0.0409 (3)	
C2	0.6827 (2)	0.35003 (13)	0.07829 (10)	0.0500 (4)	
H2	0.659051	0.287453	0.108831	0.060*	
C3	0.7392 (2)	0.33952 (15)	−0.00293 (11)	0.0563 (4)	
H3	0.750754	0.269640	−0.025395	0.068*	
C4	0.7774 (2)	0.42843 (15)	−0.04946 (10)	0.0538 (4)	
H4	0.814252	0.419332	−0.103052	0.065*	
C5	0.7981 (2)	0.63185 (15)	−0.05880 (10)	0.0520 (4)	
H5	0.836512	0.627362	−0.112434	0.062*	
C6	0.7791 (2)	0.73209 (15)	−0.02321 (11)	0.0550 (4)	
H6	0.804189	0.795738	−0.052154	0.066*	
C7	0.7214 (2)	0.73849 (13)	0.05723 (11)	0.0500 (4)	
H7	0.708614	0.806722	0.082224	0.060*	
C8	0.70289 (18)	0.54549 (12)	0.06544 (9)	0.0370 (3)	
C9	0.7607 (2)	0.53461 (13)	−0.01585 (9)	0.0426 (3)	
O1W	0.49949 (17)	0.31023 (10)	0.28209 (9)	0.0626 (3)	
H1WA	0.392446	0.331508	0.282471	0.094*	
H1WB	0.491769	0.242369	0.269682	0.094*	
O2W	0.53570 (16)	0.71260 (10)	0.24017 (8)	0.0556 (3)	
H2WA	0.480634	0.773241	0.233271	0.083*	
H2WB	0.460317	0.669091	0.258790	0.083*	
H1A	0.643 (3)	0.6562 (18)	0.1467 (8)	0.069 (6)*	
H1	0.581 (3)	0.4147 (13)	0.2114 (14)	0.086 (7)*	

### Bis(8-hydroxyquinolinium) naphthalene-1,5-disulfonate tetrahydrate Atomic displacement parameters (Å^2^)

**Table:**

	*U*^11^	*U*^22^	*U*^33^	*U*^12^	*U*^13^	*U*^23^
S1	0.0424 (2)	0.0381 (2)	0.02794 (19)	−0.00473 (12)	0.01084 (14)	−0.00284 (12)
O2	0.0634 (7)	0.0416 (6)	0.0599 (7)	−0.0036 (5)	0.0242 (6)	−0.0135 (5)
O3	0.0471 (6)	0.0540 (6)	0.0360 (5)	−0.0140 (5)	0.0142 (4)	−0.0021 (4)
O4	0.0584 (7)	0.0706 (8)	0.0291 (5)	−0.0020 (5)	0.0078 (5)	0.0066 (5)
C10	0.0356 (7)	0.0308 (6)	0.0289 (6)	−0.0024 (5)	0.0074 (5)	0.0001 (5)
C11	0.0312 (6)	0.0285 (6)	0.0300 (6)	−0.0018 (5)	0.0066 (5)	0.0002 (5)
C12	0.0317 (7)	0.0393 (7)	0.0356 (7)	0.0018 (5)	0.0102 (5)	0.0026 (5)
C13	0.0307 (7)	0.0489 (8)	0.0428 (8)	0.0052 (6)	0.0047 (6)	0.0054 (6)
C14	0.0385 (7)	0.0442 (8)	0.0309 (7)	0.0004 (6)	0.0021 (5)	0.0043 (5)
O1	0.0785 (8)	0.0460 (7)	0.0388 (6)	−0.0095 (6)	0.0159 (6)	0.0057 (5)
N1	0.0483 (7)	0.0403 (6)	0.0360 (6)	−0.0024 (5)	0.0041 (5)	0.0042 (5)
C1	0.0443 (8)	0.0437 (8)	0.0345 (7)	−0.0027 (6)	0.0017 (6)	0.0049 (6)
C2	0.0571 (9)	0.0407 (8)	0.0518 (9)	−0.0019 (7)	0.0026 (7)	0.0059 (7)
C3	0.0661 (10)	0.0474 (9)	0.0555 (10)	0.0066 (8)	0.0056 (8)	−0.0076 (7)
C4	0.0599 (10)	0.0613 (10)	0.0409 (8)	0.0071 (8)	0.0092 (7)	−0.0043 (7)
C5	0.0525 (9)	0.0649 (11)	0.0398 (8)	0.0007 (7)	0.0104 (7)	0.0136 (7)
C6	0.0582 (10)	0.0532 (10)	0.0543 (10)	−0.0050 (8)	0.0090 (8)	0.0210 (8)
C7	0.0570 (9)	0.0396 (8)	0.0532 (9)	−0.0028 (7)	0.0033 (7)	0.0076 (7)
C8	0.0370 (7)	0.0413 (7)	0.0321 (7)	−0.0018 (5)	0.0000 (5)	0.0029 (5)
C9	0.0400 (7)	0.0533 (9)	0.0344 (7)	0.0013 (6)	0.0033 (6)	0.0046 (6)
O1W	0.0647 (8)	0.0529 (7)	0.0722 (8)	0.0003 (6)	0.0171 (7)	0.0131 (6)
O2W	0.0629 (7)	0.0451 (6)	0.0618 (7)	0.0057 (5)	0.0236 (6)	0.0069 (5)

### Bis(8-hydroxyquinolinium) naphthalene-1,5-disulfonate tetrahydrate Geometric parameters (Å, º)

**Table:**

S1—O2	1.4503 (11)	C1—C8	1.414 (2)
S1—O3	1.4476 (10)	C2—H2	0.9300
S1—O4	1.4455 (11)	C2—C3	1.407 (2)
S1—C10	1.7853 (13)	C3—H3	0.9300
C10—C11	1.4314 (18)	C3—C4	1.358 (3)
C10—C14	1.3627 (19)	C4—H4	0.9300
C11—C11^i^	1.431 (2)	C4—C9	1.409 (2)
C11—C12	1.4219 (18)	C5—H5	0.9300
C12—H12	0.9300	C5—C6	1.359 (3)
C12—C13	1.362 (2)	C5—C9	1.409 (2)
C13—H13	0.9300	C6—H6	0.9300
C13—C14^i^	1.409 (2)	C6—C7	1.395 (2)
C14—H14	0.9300	C7—H7	0.9300
O1—C1	1.3433 (19)	C8—C9	1.412 (2)
O1—H1	0.819 (10)	O1W—H1WA	0.8500
N1—C7	1.3246 (19)	O1W—H1WB	0.8501
N1—C8	1.3611 (19)	O2W—H2WA	0.8498
N1—H1A	0.868 (9)	O2W—H2WB	0.8495
C1—C2	1.368 (2)		
			
O2—S1—C10	105.37 (6)	C2—C1—C8	118.63 (14)
O3—S1—O2	112.16 (7)	C1—C2—H2	119.8
O3—S1—C10	107.03 (6)	C1—C2—C3	120.36 (15)
O4—S1—O2	112.81 (7)	C3—C2—H2	119.8
O4—S1—O3	112.91 (7)	C2—C3—H3	119.0
O4—S1—C10	105.88 (6)	C4—C3—C2	121.91 (16)
C11—C10—S1	121.69 (10)	C4—C3—H3	119.0
C14—C10—S1	117.11 (10)	C3—C4—H4	120.3
C14—C10—C11	121.19 (12)	C3—C4—C9	119.43 (15)
C11^i^—C11—C10	117.91 (14)	C9—C4—H4	120.3
C12—C11—C10	123.05 (12)	C6—C5—H5	119.4
C12—C11—C11^i^	119.03 (14)	C6—C5—C9	121.12 (15)
C11—C12—H12	119.6	C9—C5—H5	119.4
C13—C12—C11	120.86 (12)	C5—C6—H6	120.4
C13—C12—H12	119.6	C5—C6—C7	119.28 (15)
C12—C13—H13	119.7	C7—C6—H6	120.4
C12—C13—C14^i^	120.62 (13)	N1—C7—C6	120.25 (15)
C14^i^—C13—H13	119.7	N1—C7—H7	119.9
C10—C14—C13^i^	120.36 (13)	C6—C7—H7	119.9
C10—C14—H14	119.8	N1—C8—C1	119.89 (13)
C13^i^—C14—H14	119.8	N1—C8—C9	119.25 (13)
C1—O1—H1	110.6 (17)	C9—C8—C1	120.85 (14)
C7—N1—C8	122.67 (13)	C4—C9—C8	118.80 (14)
C7—N1—H1A	116.7 (15)	C5—C9—C4	123.77 (15)
C8—N1—H1A	120.6 (15)	C5—C9—C8	117.43 (14)
O1—C1—C2	125.70 (14)	H1WA—O1W—H1WB	104.5
O1—C1—C8	115.67 (13)	H2WA—O2W—H2WB	104.5
			
S1—C10—C11—C11^i^	179.61 (12)	N1—C8—C9—C4	−179.40 (14)
S1—C10—C11—C12	0.00 (18)	N1—C8—C9—C5	0.5 (2)
S1—C10—C14—C13^i^	−179.89 (11)	C1—C2—C3—C4	0.8 (3)
O2—S1—C10—C11	69.66 (12)	C1—C8—C9—C4	0.0 (2)
O2—S1—C10—C14	−109.23 (12)	C1—C8—C9—C5	179.91 (14)
O3—S1—C10—C11	−49.90 (12)	C2—C1—C8—N1	−179.68 (14)
O3—S1—C10—C14	131.21 (11)	C2—C1—C8—C9	0.9 (2)
O4—S1—C10—C11	−170.60 (10)	C2—C3—C4—C9	0.1 (3)
O4—S1—C10—C14	10.52 (13)	C3—C4—C9—C5	179.58 (16)
C10—C11—C12—C13	178.45 (13)	C3—C4—C9—C8	−0.5 (2)
C11—C10—C14—C13^i^	1.2 (2)	C5—C6—C7—N1	−0.4 (3)
C11^i^—C11—C12—C13	−1.1 (2)	C6—C5—C9—C4	179.83 (16)
C11—C12—C13—C14^i^	1.5 (2)	C6—C5—C9—C8	0.0 (2)
C14—C10—C11—C11^i^	−1.6 (2)	C7—N1—C8—C1	179.67 (14)
C14—C10—C11—C12	178.84 (13)	C7—N1—C8—C9	−0.9 (2)
O1—C1—C2—C3	178.11 (15)	C8—N1—C7—C6	0.8 (2)
O1—C1—C8—N1	0.9 (2)	C8—C1—C2—C3	−1.3 (2)
O1—C1—C8—C9	−178.57 (13)	C9—C5—C6—C7	0.0 (3)

Symmetry code: (i) −*x*, −*y*+1, −*z*+1.

### Bis(8-hydroxyquinolinium) naphthalene-1,5-disulfonate tetrahydrate Hydrogen-bond geometry (Å, º)

*Cg*1 and *Cg*2 are the centroids of the C10/ C11/C11'–C13'/C14 and C11–C13/C14'/C10'/C11' rings, respectively, where primed atoms are related by the symmetry operation -*x*, 1 - *y*, 1 - *z*.

**Table:**

*D*—H···*A*	*D*—H	H···*A*	*D*···*A*	*D*—H···*A*
O2*W*—H2*WA*···O2^ii^	0.85	1.98	2.8239 (16)	176
O2*W*—H2*WB*···O3	0.85	2.02	2.8610 (15)	168
O1*W*—H1*WA*···O2	0.85	1.98	2.8150 (17)	169
O1*W*—H1*WB*···O4^iii^	0.85	2.05	2.8806 (18)	166
O1—H1···O1*W*	0.82 (1)	1.84 (1)	2.6390 (16)	165 (2)
N1—H1*A*···O2*W*	0.87 (1)	1.89 (1)	2.7347 (18)	164 (2)
C6—H6···O3^iv^	0.93	2.46	3.3153 (19)	154
C4—H4···O4^v^	0.93	2.45	3.352 (2)	165
C14—H14···O1^vi^	0.93	2.57	3.3173 (18)	137
C12—H12···O3	0.93	2.46	3.0439 (17)	121
C7—H7···*Cg*1^ii^	0.93	2.82	3.6125 (17)	144
C7—H7···*Cg*2^iv^	0.93	2.82	3.6125 (17)	144

Symmetry codes: (ii) −*x*+1/2, *y*+1/2, −*z*+1/2; (iii) −*x*+1/2, *y*−1/2, −*z*+1/2; (iv) *x*+1/2, −*y*+3/2, *z*−1/2; (v) −*x*+1, −*y*+1, −*z*; (vi) *x*−1, *y*, *z*.
